# Collimator scatter factor: Monte Carlo and in-air measurements approaches

**DOI:** 10.1186/s13014-018-1070-6

**Published:** 2018-07-11

**Authors:** A. Fogliata, A. Stravato, G. Reggiori, S. Tomatis, J. Würfel, M. Scorsetti, L. Cozzi

**Affiliations:** 1 Radiotherapy and Radiosurgery Department, Humanitas Research Hospital and Cancer Center, Milan-Rozzano, Italy; 2 PTW-Freiburg GmbH, Freiburg, Germany; 3 Biomedical Science Faculty, Humanitas University, Milan-Rozzano, Italy

**Keywords:** Collimator scatter factor, Head scatter factor, In-air output factor, Monte Carlo, Mini-phantom, Build-up cap

## Abstract

**Background:**

Linac output as a function of field sizes has a phantom and a head scatter component. This last term can be measured in-air with appropriate build-up ensuring a complete electron equilibrium and the absence of the contaminant electrons. Equilibrium conditions could be achieved using a build-up cap or a mini-phantom. Monte Carlo simulations in a virtual phantom mimicking a mini-phantom were analysed with the aim of better understanding the setup conditions for measuring the collimator scatter factor that is the head scatter component of the linac output factors.

**Methods:**

Beams of 6 and 15 MV from a TrueBeam, with size from 4 × 4 to 40 × 40 cm^2^ were simulated in cylindrical acrylic phantoms 20 cm long, of different diameters, from 0.5 to 4 cm, with the cylinder axis coincident with the beam central axis. The PRIMO package, based on PENELOPE Monte Carlo code, was used. The phase-space files for a Varian TrueBeam linac, provided by the linac vendor, were used for the linac head simulation. Depth dose curves were analysed, and collimator scatter factors estimated at different depth in the different phantom conditions.

Additionally, in-air measurements using acyrilic and brass build-up caps, as well as acrylic mini-phantom were acquired for 6 and 18 MV beams from a Varian Clinac DHX.

**Results:**

The depth dose curves along the cylinders were compared, showing, in each phantom, very similar curves for all analysed field sizes, proving the correctness in estimating the collimator scatter factor in the mini-phantom, provided to position the detector to a sufficient depth to exclude electron contamination. The results were confirmed by the measurements, where the acrylic build-up cap showed to be inadequate to properly estimate the collimator scatter factors, while the mini-phantom and the brass caps gave reasonable measurements.

**Conclusion:**

A better understanding of the beam characteristics inside a virtual mini-phantom through the analysis of depth dose curves, showed the critical points of using the acrylic build-up cap, and suggested the use of the mini-phantom for the collimator scatter factor measurements in the medium-large field size range.

## Background

The photon dose calculation for clinical radiotherapy planning is a complex process based on algorithms of different types. The current classification scheme includes the types ‘a’ and ‘b’ [1], referring to the different level of modellisation of the lateral electron transport; more recently, the classification was expanded to include the type ‘c’ [2], reserved to those algorithms where the Boltzmann equations for the electron transport is solved, either stochastically (Monte Carlo) or with an analytical approach. Even in the case of type ‘c’ algorithms, there are several factors that might affect the final accuracy of the dose calculation in different media. Among these, the precise knowledge of the dosimetric features of the clinical beams and how these are incorporated and modelled in the configuration of the algorithm (from the input to the processing of the data) can play a fundamental role and impact on the final dose calculation. In more detail, and among the various relevant factors, the field size (defined and adjusted with the collimating jaws) strongly affects the output. The output factor, OF, describes the dose variation relative to a reference field geometry, as a function of field size in certain fixed conditions. It is evaluated in a water phantom, in conditions of full scattering. Two components constitutes the output factor: the phantom scatter factor, S_p_, which quantifies the variation with field size of the scatter contribution coming from the irradiated medium and depends mainly on the beam energy; the collimator or head scatter factor, S_c_, (also called in-air output factor) which quantifies the dose variation generated by the linac head in the different geometrical conditions of varying field sizes. Some, albeit not all, of the dose calculation algorithms implemented in the clinical treatment planning systems require the measurement of the S_c_ for an accurate MU calculation, according to the configuration of the specific model. Although the concept of S_c_ and suggestions of measurements date some decades ago, its correct evaluation is still relevant and not deeply explored.

The big challenge in estimating the contribution of the linac head to the dose determination and its variation and uncertainties, is the ability/possibility of performing measurements in conditions of electronic equilibrium, while eliminating the phantom contribution, which, on the other side, is responsible of the electronic equilibrium achievement. This could be obtained by using an appropriate build-up cap added to the ionization chambers or other detectors used. This should have a sufficient thickness to guarantee the electronic equilibrium, and it must be totally encompassed by the radiation beam. For those reasons, build-up caps of high-density materials for small fields, other than plastics, have been used.

In 1991 [3, 4], the concept of the mini-phantom was proposed, and then recommended by ESTRO [5, 6]. It was conceived as a cylinder, hosting a detector, to be positioned with its axis coincident with the beam central axis. The diameter of the mini-phantom was supposed to be wide enough to ensure lateral electron equilibrium and to permit the measurement of the beam output at different depths. The possibility to place the detector at large depth allowed excluding the electron contamination, which would perturb the measurement of the head scatter factors also at depth larger than the d_max_ (depth of maximum dose in water). Since the collimator scatter factor for a test field is related to a reference field, the same phantom scatter component present in the measurements with the mini-phantom would cancel each other: from the test and the reference fields. In 1995, Li et al. [7], with Monte Carlo simulations, estimated the minimum radial thickness of a mini-phantom to reach the lateral electron equilibrium, as a function of the beam energy (with the TPR_20,10_). They concluded, for example, that the equilibrium is achieved when the radius of the mini-phantom is equal or greater than 1.3 g/cm^2^ (13 mm water equivalent thickness) for a 6 MV beam of TPR_20,10_ = 0.670. With such a thickness, the use of brass build-up caps was suggested for small field measurements. Weber et al. [8] recommended the use of brass cap, with the rule of thumb of a thickness of the cap (in g/cm^2^) at least one third of the nominal accelerating potential (in MV). This strong reduction in the thickness made the brass build-up caps suitable for small field collimator scatter measurements. However, for large fields, a small energy dependence with brass caps was shown due to an alteration of the beam spectra generated by the high-Z material, and the effect was larger for increasing beam quality. Hence, for large fields and high energies, the plastic build-up caps might be preferable.

A comprehensive and more recent report on the collimator scatter was published as a result of the AAPM Therapy Physics Committee Task Group 74 [9], also reviewing the main components of the collimator scatter factor.

Aim of the present work is the evaluation with Monte Carlo simulations of the dose generated by different field sizes from 4 × 4 to 40 × 40 cm^2^ of 6 and 15 MV beams, inside an acrylic cylinder of different diameters, mimicking a mini-phantom or build-up caps. Secondly, measurements of collimator scatter factors using different ancillary devices for in-air measurements were acquired and discussed in comparison with the Monte Carlo simulated cases. This second part aims to give a better visualization of the basic behaviour of the radiation under challenging conditions, which could help in evaluating critical situations as computed by the dose calculation algorithms implemented in the current planning systems.

## Methods

### Collimator scatter factor

The collimator scatter factor, S_c_, is defined as the following in-air measurements ratio:

$$ {S}_c=\frac{D\left( air, FS\right)}{D\left( air,F{S}_{ref}\right)} $$

where D is the dose for a fixed number of MU, FS the test field size, FS_ref_ is the reference field size, fixed to 10 × 10 cm^2^ in the current work. The measurement in air assumes the transient electron equilibrium and elimination of the electron contamination using build-up devices with adequate lateral and longitudinal thicknesses. In the current work, the ratio of the detector readings was used. This approximation was considered acceptable, since the smallest measured field size was 4 × 4 cm^2^. In the small field range (below 2 × 2 cm^2^), field size dependent corrections would have been applied.

### Monte Carlo simulations

To mimic the mini-phantom concept, different virtual cylindrical acrylic phantoms (defined in the Monte Carlo system as Lucite, with mass density 1.19 g/cm^3^) were generated, to be positioned with the cylinder axis coincident to the beam central axis. They were all 20 cm long, with diameters of 0.5, 1, 2, 3, 4 cm. Those correspond to radius of 0.3, 0.6, 1.2, 1.8, 2.4 g/cm^2^, respectively. The phantoms were created in the Eclipse treatment planning system version 13.6 (Varian Medical System, Palo Alto, USA), exported in DICOM format, and imported in the Monte Carlo environment. Square fields of 4 × 4, 5 × 5, 10 × 10, 20 × 20, 30 × 30, and 40 × 40 cm^2^ size were set with the beam axis centred along each cylindrical phantom, with a source to surface distance (SSD) of 100 cm. Monte Carlo simulations were run for a 6 MV beam generated by a Varian TrueBeam linear accelerator (Varian Medical Systems, Palo Alto, USA) for all above conditions, and for a 15 MV beam from the same linac for the phantom from 1 to 4 cm diameter.

Simulations were run using the PRIMO (version 0.3.1) package. PRIMO is a free environment for Monte Carlo simulations (http://www.primoproject.net) which allows the simulation of various clinical linacs and the radiation transport inside patient CT dataset (as well as in phantoms) to estimate the absorbed dose distributions [10]. PRIMO combines a graphical user interface and a computation engine based on the Monte Carlo code PENELOPE [11–13]. The Dose Planning Method, DPM, is a program for fast Monte Carlo simulation of coupled electron and photon transport [14], and is integrated in PRIMO and used for this study. The phase-space files, PS, for TrueBeam linear accelerators made available for research purposes by the linac vendor (Varian Medical Systems) were used for the head simulations. These PS were simulated by means of the Geant4 Monte Carlo environment, recorded and distributed in the IAEA format [15]. In the current work, the PS for 6 MV flattened beam quality, of 49.5e + 09 histories, and 15 MV flattened beam quality, of 31.2e + 09 histories were used. Inside the phantom, the transport parameters (to balance the trade-off between speed and accuracy) were predefined for DPM simulations as 50 and 200 keV for the cut-off energies for bremsstrahlung (photons) and collision (electrons), respectively. Those parameters are coded in the system and cannot be modified by the user. A calibration setting of 0.01 Gy/MU was imposed in the reference conditions (SSD = 100 cm, depth of maximum dose d_max_, 10 × 10 cm^2^ field) for both beam qualities. The simulation bin size was 0.03 to 0.23 mm in the directions perpendicular to the beam axis (depending on phantom diameter, from 0.5 to 4 cm), and 2.5 mm along the beam axis (equal to the imported phantom slice spacing resolution), according to the default DPM resolution (changeable only to a coarse 2.5 mm voxel side, too wide for the current work). The submillimetric size in two directions was generated by the virtual phantom generation in Eclipse, with a fixed matrix of 512 × 512 pixels covering a small region to host a rather small phantom diameter. A variance reduction technique (splitting in CT with a factor 300, as suggested by the PRIMO manual) was used to reduce the variance. With the use of pre-simulated phase space files, located above the collimating jaws, the absorbed doses (in Gy/MU) computed by PRIMO do not account for the radiation which backscatters to the monitor chamber. The monitor backscatter factors, MBSF, have been estimated by Zavgorodni et at [16] for the Varian Clinac and TrueBeam accelerators for all the energies available on those machines. Therefore, the collimator scatter factors estimated with the Monte Carlo in this work have been corrected for those published MBSF.

### Measurements

The in-air output factor measurements were performed using two different approaches: the build-up cap (of acrylic PMMA and brass, with relative electron densities of 1.158 and 6.975, and mass densities of 1.19 and 8.47 g/cm^3^, respectively), summarised in Table 1, and the acrylic mini-phantom. The whole equipment was manufactured by PTW, Freiburg, Germany.

**Table 1:** Build-up caps used for measurements

Type	Material	Thickness	Water-equiv. thickness	Energy range	Diameter	Name in the work
T30001.3.103	Acrylic	9.5 mm	11.0 mm	4–6 MV	26.3 mm	PMMA_11
T30001.3.104	Acrylic	13.8 mm	16.0 mm	6–8 MV	34.9 mm	PMMA_16
T30001.3.106	Acrylic	24.6 mm	28.5 mm	10–20 MV	56.5 mm	PMMA_28
T30001.3.107	Acrylic	34.7 mm	40.2 mm	20–30 MV	76.7 mm	PMMA_40
T30000.3.202	Brass	2.4 mm	16.7 mm	4–6 MV	12.1 mm	Brass_17
T30000.3.205	Brass	8.0 mm	55.8 mm	10–20 MV	23.3 mm	Brass_56

The acrylic build-up caps of 11 mm (T30001.3.103), and 28.5 mm (T30001.3.106) water equivalent thickness were set with the ion chamber both in perpendicular and parallel direction with respect to the beam axis. All the other caps were positioned only in the perpendicular direction.

The used mini-phantom (ESTRO Mini Phantom, T40036.1.010) is a cylinder of acrylic material 4 cm diameter, 18.8 cm long, and accommodates the ion chamber at a depth of 10 cm, perpendicular to the beam axis.

A Farmer-type ion chamber (PTW type 30,013, 0.6 cm^3^ sensitive volume, radius 3.05 mm, length 23.0 mm) was used for all the measurements. Three subsequent acquisitions were repeated to estimate the measurement uncertainty (the Farmer chamber noise, of less than 0.05%, was considered negligible with respect to this uncertainty and not included in the uncertainty estimation), and averaged values were collected. Most of the experiments were repeated at a temporal distance of 1 month to evaluate the stability and reproducibility of the data, and results consistent with the previous uncertainty measurements were found.

The scatter factors were measured by placing the detector at the beam isocentre, for the same field sizes used for the Monte Carlo simulations (4 × 4, 5 × 5, 10 × 10, 20 × 20, 30 × 30, and 40 × 40 cm^2^), normalised to the 10 × 10 cm^2^ field, for 6 MV and 18 MV beam qualities from a Varian Clinac DHX linear accelerator. No multileaf collimator, MLC, was used to shape the fields.

### Evaluation and analysis

From Monte Carlo simulations, depth dose curves in all simulation settings were evaluated. The doses resulting from the simulations were denoised using the iterative reduction of noise algorithm, IRON [17], implemented in the PRIMO software. This step was important due to the very small voxel size imposed by the DPM, in line with the adopted variance reduction, although this methodology could be not optimal. The depth dose data were collected as the weighted average of the depth dose curves at the central axis and ± 0.5 or ± 1 mm apart in one lateral direction. To analyse the relative characteristics of the curves, data were normalised at 5 cm depth. The lateral profiles were collected as the weighted average of two profiles on the main axes, 1 mm apart (except for the 0.5 cm diameter, where the average was on 5 profiles on each axis, 0.125 mm apart) to reduce the simulation noise. The weights were given by the uncertainty (at 2 standard deviations) estimated in each simulation point.

The dose in Gy/100MU was used to compute the collimator scatter factors at different depths and for all phantom diameters. The point dose was evaluated as a weighted average of 9 simulation points on the two main axes centred on the beam axis. The uncertainty for each collimator scatter factor was the simulation uncertainty (at 2 standard deviations) propagated for the ratio of the point dose estimation.

The measured collimator scatter factors were normalised to the 10 × 10 cm^2^ field size. Results with all build-up settings were finally compared with Monte Carlo results.

## Results

### Monte Carlo lateral profiles for different phantom diameters

**Fig. 1:**
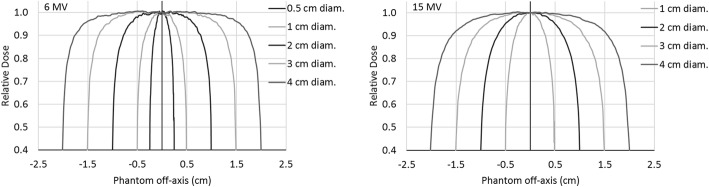
Lateral profiles for a 10 × 10 cm^2^, 10 cm depth, for the different phantom diameters. On the left: 6 MV; on the right: 15 MV

Figure 1 shows the lateral profiles for a 10 × 10 cm^2^ field, 10 cm depth, in all the analysed phantom diameters (from 0.5 to 4 cm, and from 1 to 4 cm for 6 and 15 MV, respectively). From the curves, it is possible to hypothesize that, for the 6 MV beam, the 0.5 cm, and possibly 1 cm diameter phantoms are not wide enough to guarantee lateral equilibrium, while from 2 cm diameter the presence of a small flat profile region around the central axis could suggest that the lateral equilibrium conditions are met. Similarly, the complete lateral equilibrium condition should be achieved with a 3 cm diameter for the 15 MV beam quality.

### Monte Carlo depth dose curves dependence on phantom diameter

**Fig. 2:**
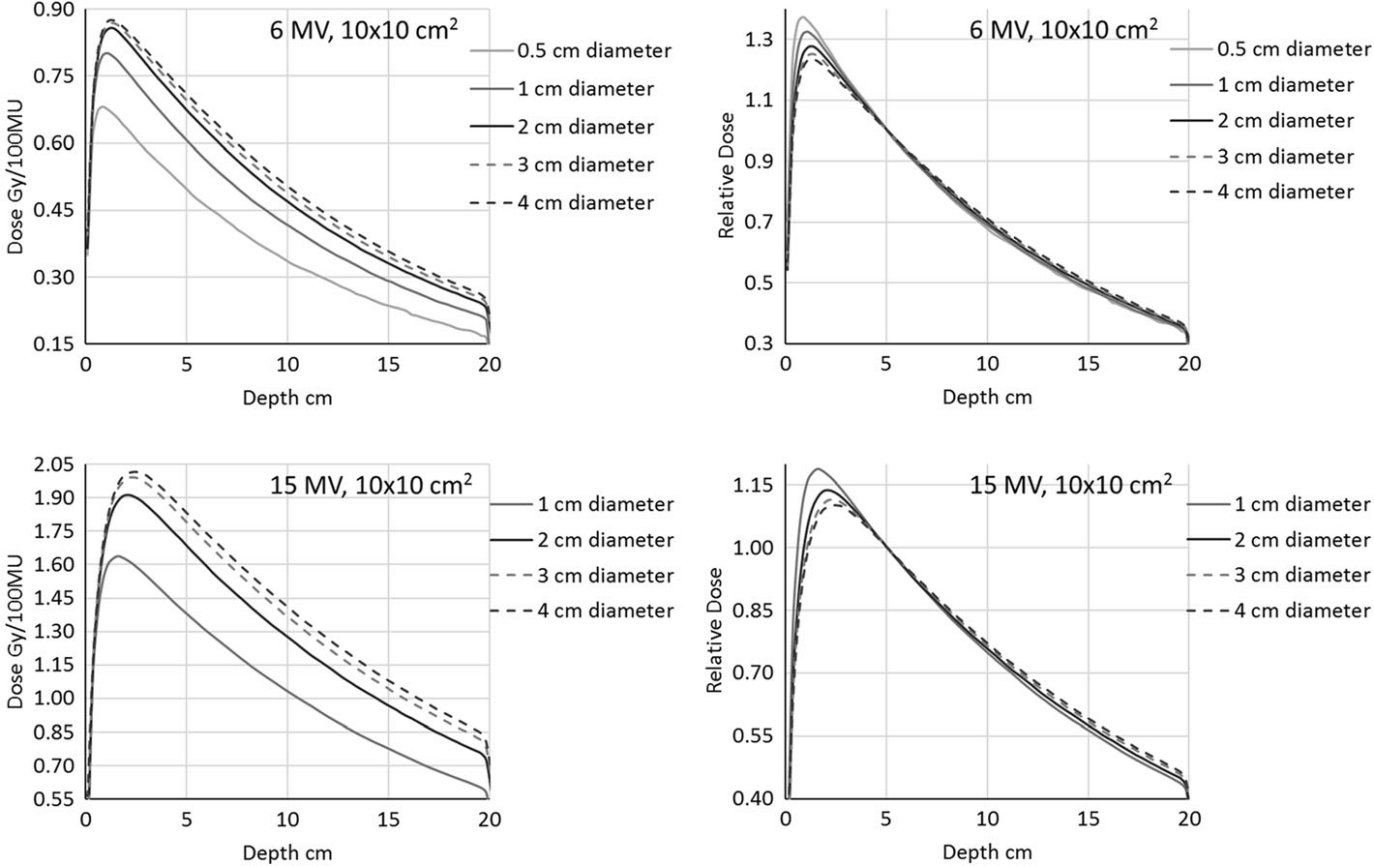
Depth dose curves for a 10 × 10 cm^2^ in the phantoms of different diameters. On the left: absorbed dose reporting; on the right: depth doses normalised to 5 cm depth. Top: 6 MV; bottom: 15 MV

Figure 2 presents a comparison of the depth dose curves for a 10 × 10 cm^2^ simulated in the acrylic cylindrical phantom from 0.5 to 4 cm diameter (1 to 4 cm for the 15 MV). Similar plots were obtained for all the other field sizes. On the left hand side of the figure, the curves are presented in terms of dose (Gy). The increasing amount of dose along the whole depth dose curve is due to the phantom scatter generated inside the cylindrical phantom of increasing diameter, as expected. On the right hand side of the figure, the same curves are reported normalised to 5 cm depth. The large variation in the build-up region is of interest: the depth of maximum dose increases with phantom diameter, and the relative amount of very low energy head scatter and electron contamination is higher for narrower phantoms.

### Monte Carlo depth dose curves dependence on field size

**Fig. 3:**
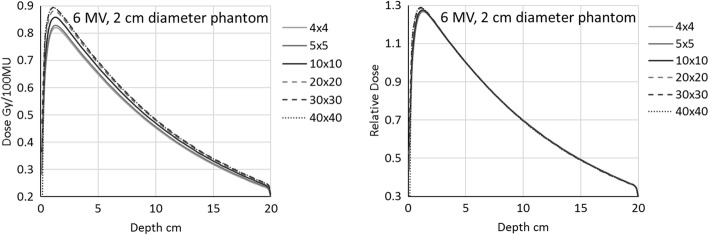
Depth dose curves for the 6 MV beam inside the 2 cm diameter phantom. Field sizes in the legend are expressed in cm^2^

Figure 3 shows the comparison of the depth dose curves of all analysed field sizes (from 4 × 4 to 40 × 40 cm^2^) of 6 MV simulated in the acrylic cylindrical phantom fixed to 2 cm diameter. Similar plots are obtained for all the other cylindrical phantom diameters. On the left hand side of the figure, the curves are presented in terms of dose (Gy). The ratio of the doses at each depth is the collimator scatter factor at that depth. On the right hand side of the figure, the same curves are reported normalised to 5 cm depth. All the curves, except in the build-up region, are almost perfectly overlapping. The small variations among curves are within the simulation uncertainty. This plot is a demonstration that the residual phantom scatter generated in the cylindrical phantom is the same for all field sizes, and the collimator scatter factor estimated in those conditions would completely cancel the phantom scatter contribution, leaving the factor to describe only the collimator scatter component. Moreover, the collimator scatter factor does not depend on the specific depth, provided the latter is sufficient to exclude differences in the electron contamination.

**Fig. 4:**
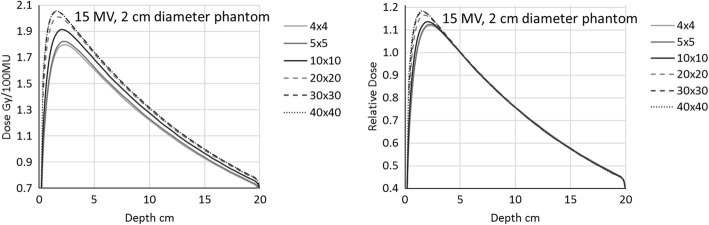
Depth dose curves for the 15 MV beam inside the 2 cm diameter phantom. Field sizes in the legend are expressed in cm^2^

Figure 4 shows the same results for the 15 MV beam quality, where the build-up dose variation due to the electron contamination is more pronounced.

### Monte Carlo collimator scatter factors

**Fig. 5:**
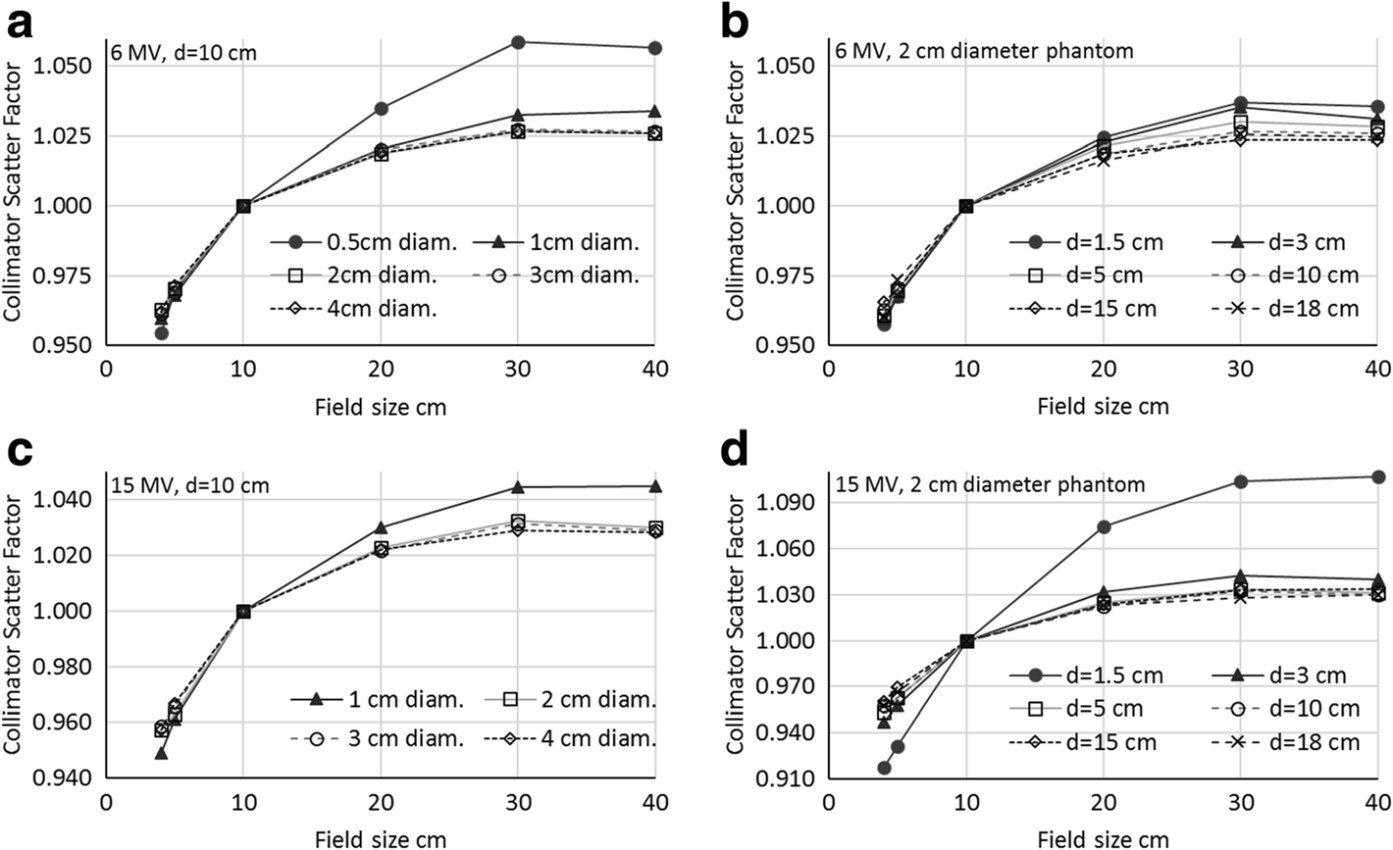
Collimator scatter factors from Monte Carlo simulation, uncorrected for MBSF: **a** 6 MV, 10 cm depth; errors at 2SD: 3.4, 1.8, 1.1, 0.9, 0.7% for phantom diameters of 0.5, 1, 2, 3, 4 cm. **b** 6 MV, 2 cm diameter phantom; errors at 2SD: 1.2% for d = 1.5 cm, 1.1% elsewhere. **c** 15 MV, 10 cm depth; errors at 2SD: 0.8, 0.7, 0.5, 0.5% for phantom diameters of 1, 2, 3, 4 cm. **d** 6 MV, 2 cm diameter phantom; errors at 2SD: 0.7%

The collimator scatter factors were evaluated at various depths and for different phantom diameters. Figure 5 reports the simulated collimator scatter factors, not corrected for MBSF, in all analysed phantom diameters at 10 cm depth, and at different depths in the 2 cm diameter phantoms. The factors remain stable for phantoms with diameter of at least 2 cm (left hand side of Fig. 5); this is an indication that narrow phantoms do not guarantee enough scatter to reach lateral equilibrium. This is also confirmed by the profiles in Fig. 1, where a flat central region was visible only from the 2 cm diameter phantom. However, collimator scatter factors are not identical for all depths (right hand side of Fig. 5): it is only for depths larger than 5 cm that the factors lie within the uncertainty. The factor variation with depth is more evident with the low energy, while for the 15 MV setting the factors result more stable when evaluated at depths larger than 5 cm.

For the small field (4 × 4 cm^2^) simulated on the 4 cm diameter phantom, i.e. for condition where the size of the phantom and the field are too close and the penumbra region falls inside the phantom, it has been noted that the collimator scatter factor increases with depth since at shallow depths the lateral equilibrium cannot be achieved. It is indeed only at large depths where the penumbra region lays completely outside the phantom, allowing a complete phantom scatter.

In summary, once an appropriate phantom diameter is used (small enough to be fully included in the beam, and large enough to ensure lateral equilibrium), the collimator scatter factor is equivalent whichever the evaluation depth, provided it is sufficient to exclude the electron contamination; 2 cm phantom diameter (or even 3 cm for high energy) and depth of 5–10 cm seem to be adequate for both beam qualities.

Table 2 reports the collimator scatter factors estimated with the Monte Carlo and corrected for the MBSF according to Zavgorodni et al. [16], for the 2 cm diameter phantom and at 10 cm depth, that is the ESTRO recommended depth for mini-phantom. Monte Carlo data have been corrected using the TrueBeam MBSF, as well as the Clinac MBSF. The first are consistent with the phase space used during the simulations, while the second are consistent with the measured data, making, with very crude approximation, an estimation of the measurement vs. simulation comparison. The differences between TrueBeam and Clinac published MBSF [16] are consistent with the same factors estimated during the beam configuration process (photon beam source model optimization) of the Acuros and AAA dose calculation algorithms implemented in the Eclipse treatment planning system (whose analysis is out of the scope of the present work).

**Table 2:** Collimator scatter factors. Monte Carlo S_c_ are corrected for the MBSF, using the published factors for TrueBeam (the original simulation), and for Clinac (according to the measurements, to compare with real measurements). Measurements (on a Clinac treatment unit) refer to Mini-Phantom data acquired with a Farmer ion chamber at 10 cm depth of PMMA

Field size	Monte Carlo		Monte Carlo		Measurements	
	S_c_ 6MV, TrueBeam MBSF	S_c_ 15MV, TrueBeam MBSF	S_c_ 6MV, Clinac MBSF	S_c_ 15MV, Clinac MBSF	S_c_ 6MV	S_c_ 18MV
4 × 4 cm^2^	0.962 ± 0.008	0.957 ± 0.005	0.961 ± 0.008	0.955 ± 0.005	0.955 ± 0.003	0.946 ± 0.002
5 × 5 cm^2^	0.969 ± 0.008	0.965 ± 0.005	0.969 ± 0.008	0.964 ± 0.005	0.968 ± 0.003	0.961 ± 0.002
10 × 10 cm^2^	1.000 ± 0.008	1.000 ± 0.005	1.000 ± 0.008	1.000 ± 0.005	1.000 ± 0.003	1.000 ± 0.002
20 × 20 cm^2^	1.020 ± 0.008	1.024 ± 0.005	1.024 ± 0.008	1.029 ± 0.005	1.026 ± 0.003	1.024 ± 0.002
30 × 30 cm^2^	1.026 ± 0.008	1.035 ± 0.005	1.033 ± 0.008	1.044 ± 0.005	1.040 ± 0.003	1.038 ± 0.002
40 × 40 cm^2^	1.027 ± 0.008	1.035 ± 0.005	1.038 ± 0.008	1.050 ± 0.005	1.050 ± 0.003	1.050 ± 0.002

### Measured collimator scatter factors

**Fig. 6:**
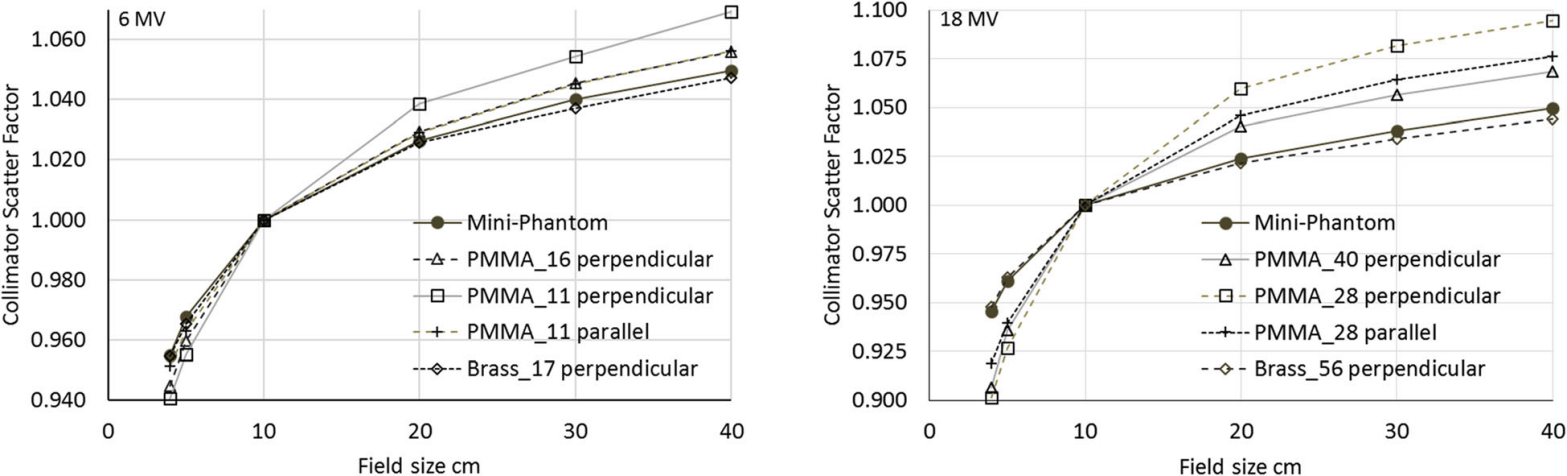
Measured collimator scatter factors. The Mini-Phantom has 4 cm diameter, and measurements were acquired at 10 cm depth of PMMA. Build-up caps were of PMMA and brass material, and had water equivalent thicknesses in mm according to the legend, in perpendicular or parallel setting relative to the beam axis

The different solutions adopted to measure the collimator scatter factors generated different results. Figure 6 shows the measurements acquired with the mini-phantom, the acrylic cap (two thicknesses per each energy, and two orientations for the thinner of the two), and the brass cap.

For both energies, the thin acrylic cap with its axis positioned perpendicular to the beam axis resulted in an overestimation of the factors for large fields, and an underestimation for smaller fields. The same acrylic cap parallel to the beam axis showed similar factors as larger caps perpendicularly positioned; for the 6 MV the 11 mm water equivalent thickness cap positioned parallel to the beam gave almost identical factor than the 16 mm perpendicularly placed. Similarly, for the 18 MV, with the parallel 28 mm and the perpendicular 40 mm water equivalent thickness caps. However, all the acrylic caps showed a too large variation of the collimator scatter factors with field sizes. The brass and the acrylic mini-phantom showed on the other side very similar results, with the brass presenting slightly less variation with field size relative to the mini-phantom.

Those results could suggest that the acrylic caps would probably need larger thickness, especially in the direction of the beam. Secondly, the perpendicular setting, having lost the cylindrical symmetry, might generate an unwanted amount of phantom scatter, which depends on the field size.

Table 2 reports the collimator scatter factors measured in the mini-phantom at 10 cm depth.

## Discussion

Collimator scatter factors have been evaluated in this work through Monte Carlo in order to better understand the beam characteristics in a phantom mimicking the mini-phantom concept. The same factors were measured using different build-up caps and a mini-phantom.

The factors measured in the current work using the mini-phantom resulted coherent with the data reported by the AAPM Report of the Task Group 74 in their appendix [9] within the 0.5% estimated uncertainty. Similarly, the factors here measured with the brass build-up cap were fully compatible with the published data [9].

Li et al. [7] investigated the problem of the mini-phantom minimum diameter. In their work, with Monte Carlo simulations, they estimated the minimum radius needed to achieve the lateral electron equilibrium. They concluded that when the mini-phantom radius is very small, the contaminant electrons generated outside are not completely absorbed, and significant changes in the collimator scatter factor values can be observed. A linear relationship between TPR_20,10_ and the radius to achieve the lateral electron equilibrium was found. The suggested minimum radius was given in terms of areal density (thickness multiplied by the mass density) as 1.3 and 1.9 g/cm^2^ for 6 and 15 MV beams, respectively, for data obtained at 5 cm water equivalent depth. They noticed that the reported relationship is not applicable to high-Z material, as the case of brass build-up caps. Differently, Jursinic et al. [18], with experimental measurements acquired at 10 cm depth, found lower values for the lateral electron equilibrium, reporting a minimum areal density of 0.7 and 1.0 g/cm^2^ for the 6 and 18 MV beams, respectively. Our data, from Monte Carlo simulations, although not aiming to find the minimum phantom radius, showed a possible complete lateral electron equilibrium for the phantom setting of 2 cm diameter for 6 MV, and 3 cm for 15 MV (these have not to be read as minimum phantom size). These values, in terms of radius expressed as areal density, are 1.2 and 1.8 g/cm^2^, respectively, in full accordance with the Li results. Conversely, the Jursinic data would consider sufficient a corresponding phantom diameter in our work of 1.2 and 1.7 cm for the low and high energies, respectively. This cannot be fully confirmed by our results, having simulated only 1 and 2 cm diameters with no better refinement, being out of the scope.

Another important factor influencing the head scatter and its estimation is the electron contamination, which in principle should be excluded from the collimator scatter factor. It is however known that the electron contamination is still present and not negligible at depths larger than the d_max_. This is one of the reasons leading to the mini-phantom introduction, since it is possible to measure the output at different depths, keeping minimal the lateral scattering. Venselaar [19] presented a formalism including the electron contamination, and reported related measurements. They found, as an example, for a 40 × 40 cm^2^ from a 6 MV beam from a Saturne GE linear accelerator, an electron contamination of about 2% estimated at 2 cm relative to 10 cm depth. In our work, the difference between collimator scatter factors simulated at 1.5 and 10 cm depth for the 6 MV beam, once in conditions of complete lateral electron equilibrium, of 1%. The two results cannot be strictly compared, since the electron contamination depends on the linac, and in the two works, different machines were used. However, it is clear that a rather high depth has to be adopted in the mini-phantom to avoid electron contamination. Frye et al. [20], who directly measured in conditions where the electron contamination was cancelled by using an electromagnet to deviate the contaminating electrons from the beam, already proved this. They found that at 10 cm depth there is no more contamination.

Both the measuring depth in the mini-phantom and the electron contamination issues have been easily visualised in the current work with the Monte Carlo simulation and the depth dose curves evaluation. This different approach with respect to the published data, confirms the constancy of the beam penetration in the cylinder when varying the field size, and the large difference in the build-up region (Figs. 3 and 4, right). The independence of the depth doses from the field sizes is a confirmation that the commonly seen variation of the depth dose curves with field sizes is due to the lone phantom scatter. This also demonstrates that, provided a depth sufficient to exclude the electron contamination, the collimator scatter factors should not depend on the measuring depth. In addition, from both the depth doses as shown in Fig. 2 (right), and more clearly the collimator scatter factors with different phantom diameter in Fig. 5 (left), we confirm that the lateral electron equilibrium has to be complete to have an accurate S_c_ estimation.

The results from Monte Carlo data in terms of collimator scatter factor should be carefully evaluated. Only in the case where the radiation backscattered to the monitor chamber is modelled explicitly, the resulting S_c_ can be considered as consistent values. However, this is possible only when the treatment head geometry is available, that was not the case of the current study. For that reason, the S_c_ from our simulations were corrected according to the published monitor backscatter factors [16] to be compared with measured data. To note, from the Zavgorodni et al. results, the correction is not negligible also for very large fields, and not identical for all linacs. For example, the reported MBSF for a 40 × 40 cm^2^ of 6 MV was 1.014 and 1.003 for a Clinac and a TrueBeam, respectively; the same figures for high energies were 1.020 and 1.006 (18 and 15 MV, respectively).

From the measurements of the current work, the tested acrylic build-up caps presented insufficient thickness. In particular, when the cap has its axis perpendicular to the beam axis, the electron equilibrium as well as the geometrical conditions are not adequate. The parallel setting should be preferred. The devices that had better fulfil the expectations are the mini-phantom and the brass cap. This last one, due to the high-Z material, could influence the reading for large fields and high energies [8]. The mini-phantom gives hence the best compromise for collimator scatter factor measurement in all conditions. However, a setting with the ion chamber axis parallel to the beam axis could be preferred to the perpendicular setting, as it was in the current work. The parallel setting keeps the cylindrical symmetry, allowing a constant lateral thickness, which assures the same lateral electron equilibrium in all the directions, and more consistent results due to the integration volume position.

A limitation of the current work is the collimator scatter factor evaluation only for large fields. The small fields were out of the scope, since in those cases a completely different approach has to be considered, using caps or mini-phantoms forcedly of high-Z materials. In those cases, also the correct estimation of the MBSF has to be carefully considered for Monte Carlo simulations.

Another important limitation is the methodology applied to the Monte Carlo settings, in terms of voxel size (forcing the use of a strong variance reduction and the IRON denoising procedure), and in terms of energy cut-off for electrons (collisions) of 200 keV, imposed by the system, while a lower value could probably better estimate the build-up and lateral equilibrium impact.

As a final remark, the measurement of the collimator scatter factor is still a currently open topic for what concerns the difficulty in its proper measurement and its use in some dose calculation algorithms. Although in the past its importance was linked directly to the dose calculation algorithms based on TMR (tissue-maximum ratio), again today it is fundamental to have a good knowledge of all the dosimetric aspects. The current dose calculation algorithms are mostly based on analytical descriptions of the beam. It is hence the correct and accurate acquisition of the beam data, together with the basic formalism knowledge of the beam modelling that could allow a safe use of the advanced algorithms in all clinical conditions. With the increasing use of dose escalation, hypofractionation schemes, stereotactic treatments delivered with advanced techniques (intensity modulation and volumetric modulated arc therapies), there is an increasing demanding request of accuracy. The need of an accurate knowledge also in the small field frame is the next step of our project, which was considered out of the scope in the current study.

A deeper knowledge of the beam behaviour in conditions close to the electron equilibrium/disequilibrium boundary is an important milestone also for decision processes like the choice of the reference condition of the linear accelerators, or relative dosimetric data normalization for the algorithm beam configurations, or again the choice of the reference conditions for setting the absorbed reference dose in the treatment planning systems.

## Conclusion

A better understanding of the beam characteristics inside a virtual mini-phantom through the analysis of depth dose curves, showed the critical points when using the acrylic build-up cap, and suggested the use of the mini-phantom for the collimator scatter factor measurements in the medium-large field size range.

## Abbreviations

d_max_: Depth of maximum dose in water
DPM: Dose planning method
MBSF: Monitor backscatter factor
OF: Output factor
PS: Phase space
S_c_: Collimator scatter factor (also called head scatter factor, or in-air output factor)
SD: Standard deviation
S_p_: Phantom scatter factor
SSD: Source to surface distance
TMR: Tissue-maximum ratio
TPR: Tissue-phantom ratio
